# Spices and Seasoning Mixes in European Union—Innovations and Ensuring Safety

**DOI:** 10.3390/foods10102289

**Published:** 2021-09-27

**Authors:** Maria Śmiechowska, Joanna Newerli-Guz, Magdalena Skotnicka

**Affiliations:** 1 Department of Quality Management, Faculty of Management and Quality Science, Gdynia Maritime University, 81-225 Gdynia, Poland; m.smiechowska@wznj.umg.edu.pl (M.Ś.); j.newerli-guz@wznj.umg.edu.pl (J.N.-G.); 2 Department of Commodity Science, Faculty of Health Science, Medical University of Gdańsk, 80-210 Gdańsk, Poland

**Keywords:** spices and seasoning mixtures, product innovation, food safety

## Abstract

Spices are an important group of food products of great importance in nutrition and food technology. They are mainly used to shape the sensory properties of food in gastronomy, in home cooking, and in industry. Ensuring quality and safety is one of the basic tasks of spice producers. The aim of this review is to present the threats to the consumer related to the presence of spices and seasoning mixes in the diet. Therefore, special attention was paid to such risks as excess sodium chloride (and sodium) in spice mixtures, the use of additives influencing the sensory experience, and irregularities in the labeling of spices and seasoning mixes for the presence of additives and allergens. The threats regarding microbiological safety and the presence of heavy metals, pesticides, plant protection products, as well as synthetic fertilizers and undeclared additives are also presented and the issue of adulteration and lack of authenticity of spices and spice mixtures is discussed. Using data from IJHARS planned inspections and notifications registered in the EU Rapid Alert System for Food and Feed (RASFF) for 2015–2019, as well as the results of own research, an analysis of the risks caused by herbs and spices was carried out. Strategic activities of companies producing spices focus, among others, on improving production and expanding the commercial offer with new, attractive products. The article reviews product and process innovations in spice mixes and the methods of ensuring safety in this group of food products.

## 1. Introduction

Spices are a group of food products with very diverse composition and effects. There are many definitions of spices in the literature. Most scientists define spices as products of plant origin, although they may have a more complex composition, as is the case with seasoning mixes.

Spices have been used in nutrition and medicine since ancient times. Currently, this group of food products is gaining importance due to the intensive research on ingredients and the search for new bioactive compounds that can be used as modern medicinal agents and functional food ingredients and in current cosmetology, which aims to limit synthetic ingredients in cosmetics [1,2]. Spices may also have additional properties, e.g., they can have a positive effect on the human body and preserve food [3].

In recent years, there has been a significant development especially in the group of spice mixes. Spice mixes have become a convenient spice group that is undergoing the most innovative changes. This development has been influenced by many factors, such as the increase in consumer awareness of the role and importance of spices and the development of tourism.

The aim of this work is to present the innovations that have taken place in spices and seasoning mixes. The literature in this area was reviewed and attention is drawn to the risks pertinent to spices and seasoning mixes. The rapid development in this group of food products results, on the one hand, from the growing market demand and, on the other hand, from the competitiveness of spice producers directed not only at individual consumers but also at the food industry.

## 2. Spices and Seasoning Mixes—Definitions and Importance in Food and Nutrition

Codex Alimentarius enumerates substances added to food to enhance the aroma and flavor and includes salts, spices, soups, sauces, salads and protein products. They are classified as salt and salt substitutes, herbs, spices, seasonings and condiments, vinegars, mustards, soups and broths, sauces and similar products, salads and sandwich spreads, yeast and similar products, soy-based spices, and finally protein products from sources other than soy. The concept of spices is thus broader, as it includes not only spices such as plants and/or parts thereof, but also salt, bouillon cubes, soy sauce, fish sauce, or ketchup, all used to improve palatability [4].

The authors of this study draw attention to a significant difference in the requirements that are imposed on herbal raw materials intended for medicinal use on the one hand, and herbs intended for gastronomy, culinary purposes, and food processing on the other hand. Herbs for medicinal purposes must meet the same quality and safety standards as are set for medicines. On the other hand, the herbal raw material intended for the food industry or catering must meet the requirements for all food products. Many researchers dealing with spices do not pay attention to this issue at all, and in our opinion this is a fundamental difference, because it is difficult to expect from a herb or spice grown in a pot or home garden that it will meet the requirements of a therapeutic agent [5].

Two kinds of spice mixes can be distinguished—the so-called blends and the seasonings. *Blends* are composed only of the appropriately selected herbs and spices. This requirement is met, for example, by Provencal herbs that are among the oldest European seasoning mixes with a well-defined composition [6]. The second group is *seasonings*, in which the spice mix, apart from components of plant origin, includes other ingredients such as salt, monosodium glutamate, and citric acid. Nowadays, many more additional ingredients are included in seasonings.

Spice mixes are composed for reasons such as:

1. the desire to obtain stronger, more refined, or repeatable tastes in dishes;
2. the need to streamline the preparation of meals in gastronomy and at home;
3. the need to ensure the quality of spices by selecting the proper ingredients, i.e., the inclusion of individual spices and their proportions;
4. the standardization of the composition of the spice mixtures in order to obtain repeatability of sensory impressions in the prepared dishes in gastronomic conditions and in production.

Spice mixes can be produced in various forms and the most common are as follows:

1. loose spices of varying degrees of fragmentation–cut, broken, or powdered;
2. liquid spices, emulsions, or liquid extracts;
3. spices in the form of pastes or dry extracts.

## 3. Risks Related to the Use of Spices and Seasoning Mixes

Herbs and spices are used for their properties which result from their rich composition. Many works have been devoted to the use of herbs and spices in medicine and cosmetology [7,8,9,10,11]. It should be noted, however, that herbaceous plants and spice raw material used in food processing and in the production of spices can pose a threat to the life and health of consumers. This may result from various factors, including the natural toxicological aspects of plants, as well as threats that may arise during technological processes or during storage and distribution.

### 3.1. Health Risks Related to Excess Sodium Chloride in Food

A substance that is often found in the composition of spice mixtures as the main component is table salt. Consumers are primarily interested in purchasing spice mixes and not attention is not always drawn attention to their composition. Some of the spice mixes may contain significant amounts of table salt and constitute a health risk when used excessively [12]. Consumers with a dietary salt restriction recommendation should check the composition of spice mixes. According to the above researchers, the salt (sodium) intake repeatedly exceeds the permissible limits and the need to reduce its consumption is apparent. Many ready-to-eat products, including convenience foods, are a hidden source of salt [13].

In food processing, especially in meat, fish, and vegetable products, large amounts of salt, including sea salt, are used. Sodium is an element that performs important functions in the human body, but its excess leads to hypertension and impairs the functioning of the kidneys and the cardiovascular system [14,15,16,17]. In many countries, actions are being taken to reduce the dietary intake of table salt (sodium). Many scientific societies, and the World Health Organization, are calling for a reduction in sodium intake [18]. There are also controversial opinions on this subject [19]. In addition, recent studies have shown that sea salt can be contaminated with micro and nanoplastics, and the presence of these compounds raises concerns due to their possible penetration through the food chain [20].

### 3.2. Health Risks Related to the Addition of Flavor Enhancers to Spices

Another substance commonly included in spice mixes is sodium glutamate. This compound is added for its taste enhancing action, responsible for the formation of *umami* flavor. Sodium glutamate is often used in Chinese cuisine and is responsible for the formation of a broth taste, characteristic of Chinese soups and concentrates. It is also infrequently responsible for allergic reactions, manifesting by a hot sensation in the mouth, itchiness of the skin, and sometimes atopic changes and increased perspiration [21,22,23].

Not only sodium glutamate, but also inosine 5′-monophosphate (IMP) and guanosine 5′-monophosphate (GMP) are responsible for the enhancement of the *umami* flavor. New research in this field is revealing *umami* perception mechanisms and the effect of umami on satiety, as well as peripheral and central *umami* coding. Recent studies have revealed the individual *umami* taste perception and its relationship to genotype, as well as the presence of *umami* taste receptors in the gastrointestinal tract and interactions between the brain and the gut [24].

### 3.3. Health Risks Related to the Presence of Allergens in Spices and Their Labeling

The presence of allergenic substances in food, including spices, causes concern and the need to take action to warn against them and investigate the possibility of eliminating them. In order to reduce occurrence of allergic reactions after eating meals seasoned with spice mixes, producers of spices, as well as other processed foods, are required to label them in accordance with Regulation (EU) No 1169/2011 of the European Parliament and of the Council of 25 October 2011 on the provision of food information to consumers. The list of substances or products causing allergies or intolerances is included in Annex II to this Regulation. It lists 14 groups of products, most of which may be included in seasoning mixtures. There are no quantitative criteria in the European Union for allergen alert labeling in food [25]. Such criteria were introduced on the basis of national regulations in Switzerland and Japan [26]. 

All kinds of soy sauces and fish sauces, sauces with added shellfish, vegetable sauces with celery, mustard seeds as a component of mustard, and many mixtures added to meat preserves and dishes, can cause allergic reactions or anaphylactic shock following unaware consumption. Tourists who consume street food and have no knowledge about the ingredients of the dish may be particularly exposed to this type of risk [27].

Tomatoes are an ingredient of many spices, but are primarily used in the production of ketchups and sauces. They can also cause adverse allergic reactions in susceptible people. However, recent studies have shown that organically grown tomatoes seem to have an advantage over conventionally grown ones in terms of reduced allergenic potential [28]. 

Herbaceous plants and spice raw material can also be an allergenic hazard. The process of drying and composing spice mixes does not fully remove plant pollen and hairs covering the leaves of tomentose plants, which are irritating and may cause allergic reactions, the effects of which can be very serious for the consumer. Likewise, fresh herbs and flowers can cause allergic reactions due to the presence of pollen and irritating hairs.

A number of unpleasant sensations in hypersensitive people can be caused by spices such as pepper or chili that contain irritating substances. High concentrations of piperine or capsaicin can lead to many adverse gastrointestinal symptoms as well as neurological reactions including pain [29]. The content of capsaicin and dihydrocapsaicin is the basis of the Scoville scale, which determines the hotness of a product or spice [30]. 

Therefore, most spices available on the market include relevant warnings regarding the content of allergenic products or even the possibility of containing trace amounts of a substance that could result in an allergic reaction. This type of information appearing on the packaging of spices also protects the manufacturer against the occurrence of health hazards in consumers and against possible litigation.

### 3.4. Adulteration and Lack of Authenticity of Spices and Seasoning Mixtures

The problem of adulteration of spice mixes may concern, among others, changes in the quantity and quality of ingredients, the use of a raw material devoid of a bioactive substance as a result of, e.g., distilling an essential oil, which is a valuable raw material for the production of perfumes and other cosmetics, or adulteration of the country of origin of the spice. Information about the origin of the raw material should absolutely be given for single-ingredient spices, but unfortunately this information frequently cannot be found on the packaging. For some spices, information about the origin is very important due to the differences in the quality and price of spices coming from different cultivation regions. This information is especially important if the spice comes from the natural state, it is then very important to indicate the area where the raw material is obtained. Identification of adulterations in herbs and spices is often very difficult and requires the application of advanced analytical methods [31,32,33,34,35].

For example, cinnamon exists in two popular forms, as Ceylon cinnamon or true cinnamon (*Cinnamon zeylanicum* Blume), which is grown in Sri Lanka and southern India, and as cassia (*Cinnamon aquaticum* Ness), which is grown in China, Indonesia, and Vietnam. This spice is most commonly marketed as ground cinnamon or in spice mixtures for baking cakes and other preserves. Since ancient times, it has been used in folk medicine as a therapeutic agent with antipyretic, anti-inflammatory, and antibacterial properties. Ceylon cinnamon (*C. zeylanicum* Blume) has a stronger effect and is additionally characterized by a much more pronounced, sweet aroma, unlike cassia [36]. In Europe, the demand for Ceylon cinnamon is growing particularly fast, as it has recently been gaining popularity due to the discovery of its new medicinal properties. Hence, the number of adulterations of this spice with cassia variety has increased significantly. Identification and testing for authenticity is a difficult matter and requires extremely advanced analytical methods [37,38]. Therefore, in recent years, more and more attention has been paid to non-destructive methods of monitoring the quality and safety of spices [39].

In 2018, as a result of analyzes carried out by the IJHARS (Agricultural and Food Quality Inspection in Poland) laboratories, 16.5% of tested herbal spice samples were questionable from the point of view of organoleptic assessment. In turn, the share of rejected batches of herbal spices (within scheduled monitoring) on grounds of non-compliance of physicochemical parameters with the declared ones was 8.3%, while the share of questioned batch labeling was as high as 26%. Consequently, every fourth sample of spices was incorrectly labeled [40]. 

A large number of food-safety-related incidents in 2008–2018 was noted by Soon et al. [41]. The total number of specific incidents related to food safety and/or market withdrawals with known or suspected causes found, in 2008–2018, to be 2932. Among others, they identified that the main cause of incidents was the content of non-declared allergens which led to withdrawal of products from the food market.

### 3.5. Threats Connected with the Microbiological Safety of Spices and Seasoning Mixes

The information from RASFF reports in the food category “Herbs and Spices” is particularly disturbing. From a detailed analysis, it appears that in the period from 1 January 2015 to 31 January 2019 there were 799 alarm notifications, notes, and border rejections of products in this food category [42]. Many of these products are included in spice mixes or are ready-made spice mixes. An increasing number of notifications in 2018–2019 is worrisome, especially pertaining to herbs and spices already present on the market (Table 1).

A detailed analysis allowed us to distinguish the main reasons for the notifications that are included in Table 2, which showed that notifications related both to single spices and spice mixes.

The spices that were most contaminated with aflatoxin B1 included chili and various spices mixes, mainly from Asian countries. There were as many as 220 such notifications in the RASFF database in 2015–2019. 

Over 200 cases of notifications were reported due to the presence of *Salmonella* sp. in various spices and blends. Several dozen notifications regarding the presence of *Salmonella* sp. in black pepper from Brazil drew attention.

The situation in terms of microbiological purity and the presence of other impurities in spices did not improve. As a consequence, in 2013 the FDA issued a draft risk profile “FDA Risk Profile: Pathogens and Filth in Spices”, which aimed to improve the quality of spices.

The problem of microbiological contamination of food, including herbs and spices, is discussed by many authors [43,44,45]. The increasing number of notifications concerning the presence of *Salmonella* sp. in spices and seasoning mixes available on the European market is disturbing. In 2015–2019, the largest number of these notifications was recorded on the British (170), German (123), Dutch (115), and Spanish (66) markets. Fewer notifications were reported by other European countries, such as Belgium (34), Finland (38), France (32), and Italy (30) (RASFF-EC-Notifications list). Pigłowski [46] also draws attention to the increase in the number of notifications regarding food hazards on the European market. Analyzing the list of RASFF notifications in 2011–2014, he found a high number of food notifications from Italy, Germany, France, and the United Kingdom, with the most common distribution status in the notification being “distributed on the market (possible)”.

The use of radiation treatment in spices, mainly from China, was undeclared five times. This problem is also analyzed and noticed by control authorities, as well as in the studies of various authors [47,48,49].

### 3.6. Threats Due to Heavy Metals, Pesticides, Plant Protection Products, Synthetic Fertilizers, and Undeclared Additives in Spices

Table 3 presents the risks related to the presence of plant protection products, pesticides, and undeclared additives in spices. Various crop protection chemicals and pesticides have been identified more than fifty times in fresh, dried, and powdered spices. Such dangers may already appear at the stage of obtaining the raw material. Many authors draw attention to the contamination of plant products with chemicals, including those used during plant vegetation. Such substances include heavy metals, pesticides, plant protection products, fertilizers, and other substances used in plantations [50,51,52,53].

The RASFF notifications show that despite the obligatory declaration of additives used in the processing of spices, producers did not provide information about the presence of coloring agents or the addition of sodium benzoate. These additives were used in Asian and Middle Eastern countries in very popular spice mixtures (Table 3).

In recent years, the demand for edible flowers, which are often used as a fragrance and visual additive in spices, has increased significantly. Unfortunately, edible flowers are often contaminated with pesticides and do not meet microbiological standards. Information proving the lack of proper supervision over the production of flowers intended for consumption is not overwhelming, and it has even been shown that flowers intended for bouquets can be eaten [54].

## 4. Product and Process Innovations in Spice and Seasoning Mixes

Recently, an intensive development of product and process innovations in spices, especially in seasoning mixes, has been observed. Spice mixes are of great importance in industrial food processing and in catering technology. However, if they are improperly prepared, composed, or stored, they can pose a risk to consumers. Spice mixes can be treated as convenient additives for the preparation of meals. They standardize the sensory values of dishes and facilitate the work of preparing meals at home, as well as in canteens and restaurants.

### 4.1. Product Innovations in the Group of Spices and Seasoning Mixes

It can be noticed that the innovation potential in this group of products consists in creating spice mixes for individual dishes, such as gyros or bruschetta spice, and in creating national mixes such as Greek herbs, Italian cuisine spices, or Chinese spices, as well as regional seasoning, such as Tuscan seasoning, Mediterranean cuisine herbs, or Balkan spices [55].

New trends in spice mixes lead in several directions. The first one is to eliminate the non-plant elements to a greater extent. Spice mixtures without added monosodium glutamate and with a reduced salt and sugar content have begun to appear on the market. Many manufacturers have also stopped adding ingredients containing gluten and mixtures of spices replacing table salt have appeared on the market [56,57].

The increasing consumer awareness has prompted the producers of spices to react to signals coming from the market and to introduce product innovations in spice mixes; the search for novel natural flavor enhancers is also ongoing [58]. 

Information on packaging also informs consumers about the possibility of the presence of trace amounts of other allergens, such as chicken eggs, celery, soybeans, and charlock. Information of this type is placed on the spice packaging and serves not only marketing purposes, but also has informational potential. Sometimes, however, this information is unreadable and consumers, especially older ones, are not able to read it.

The information efficiency of spice packaging is increasing, but, according to the IJHARS reports, many producers continue to show deficiencies in this regard [40].

Safety and quality of the prepared dishes are crucial in gastronomic production. According to the Regulation (EC) No 852/2004 of the European Parliament and of the Council, food producers, including catering establishments, have full responsibility for food safety and must ensure that it is guaranteed [59]. System solutions help in the production of food of good, repeatable quality that is safe for the health of the consumer. The need for information is also increasingly noticed by restaurateurs who frequently include a list of allergens present in the dish in their menus [27].

As shown in Table 2, the share of notifications questioning the microbiological status of herbs and spices is significant. It is postulated that one of the reasons for the low microbiological quality is non-compliance with the principles of good hygienic practice after harvesting the raw material, especially during the drying process of herbs and spices [60,61]. Hence, the solution to this problem is seen in new, innovative methods of preserving the raw material for the production of spices [62].

In the EU, the SPICED project (“Securing the spices and herbs commodity chains in Europe against deliberate, accidental, or natural biological and chemical contamination”) has developed systems to detect and neutralize contaminants in herbs and spices, with innovations including chemical fingerprinting methods, chemometrics, and decontamination systems [63].

As environmental awareness grows, so too does the demand for organic herbs and spices. The greening of the production of herbs and spices goes in two directions, one concerning innovative packaging solutions for this group of food products, and the other concerning the obtained raw material. Spice packaging is increasingly often made of paper or glass. The labeling also includes information on the rules for handling the packaging after the product is used. On the other hand, there is a growing demand for herbs and spices from organic sources. The raw material for their production may be harvested in the natural state or come from organic farming. Spice producers ensure that the raw material for the production of spices comes from the purest crops, but the number of certified spice plantations is not high. The production of organic herbaceous material is unable to meet the demand and this also applies to spices, as the supply of organic raw material is small [64].

### 4.2. Process Innovations in the Group of Spices and Seasoning Mixes

Conventional methods of obtaining raw material for the production of spices are also being replaced by new, alternative methods, which leads to reductions in the level of risk, mainly microbiological, by introducing novel methods for decontamination of herbs and spices. A wide range of physical methods have been proposed, such as supercritical carbon dioxide extraction, high pressure technology, ionizing and infrared radiation, microwaves, extrusion, and superheated steam treatment. However, not all of the proposed methods have gained public approval, in particular those that use radiation. The effectiveness of the methods also varies [65,66,67,68]. Chemical methods have also been applied in the decontamination of herbs and spices. Chemicals such as methyl bromide, formaldehyde, ethyl alcohol, and ethylene oxide are used. Currently, the application of these compounds for microbial inactivation is being abandoned because they affect the content of bioactive substances in herbs and spices. The use of formaldehyde is an extremely effective technology for chemical decontamination of herbs, but nowadays it is no longer used because of a significant reduction in the amount of essential oils in plant materials and the bad influence of human health. The increasing level of formaldehyde in the body may play an important role in the development of Alzheimer’s disease [69]. Residues of formaldehyde can be detected in different products and these appear to be derived from the use of formaldehyde as a feed preservative. The EU has now prohibited the use of formaldehyde as a feed preservative [70]. However, this method is quite widespread outside Europe and is used by the largest manufacturers of herbs [71] due to the low cost of its use.

In Europe consequently, ozonation is becoming increasingly used. Ozone decontamination allows for obtaining products that meet the requirements of an innovative product A special fumigation with ozone gas can be used as a suitable method for achieving microbiological stability in herbs [72,73]. Another alternative decontamination method is extrusion. However, this method also has limitations and cannot be used to sterilize every raw material [62]. 

In the hitherto innovative solutions in the herbs and spices market, product innovations have dominated. The latest trends indicate that process innovations are also being introduced in this category. A novel technological approach is microencapsulation consisting in enclosing extracts and essential oils of herbs and spices in special envelopes. Both the methods of forming microcapsules, as well as the raw materials from which the shells are formed are innovative [74]. Microencapsulation is used to maintain the quality of bioactive compounds or to increase their suitability for use in food, nutraceutical, or cosmetic products. Microencapsulation and nanoencapsulation are the two main methods in encapsulation technology, both of which have a particular specialization in improving product functionality. There has been great interest in nanoencapsulation, which can be one way to deliver bioactive compounds due to high encapsulation efficiency, increased bioavailability, better stability, sustained release profile, and masking of unwanted tastes in products. Microencapsulation also ensures the standardization of the concentration of bioactive compounds, as they can vary in plants depending on geographic, seasonal, and processing factors. Another application noticed by breeders interested in the greening of the environment and animal husbandry is the use of microcapsules of bioactive herbal and spice compounds in feed for microbiological protection of animals instead of using antibiotics. This could at least partially reduce the use of antibiotics [75]. Antibiotic contamination of land and sea waters is increasing due to the development of aquaculture, as evidenced by research results [76,77].

## 5. The Role of the Law in Advancing Innovation and Ensuring the Quality of Herbs and Spices

Innovative projects in the field of food economy should bring solutions that are safe and beneficial to human health and also benefit the environment. In order to improve the global quality of spices, the FAO/WHO Codex Alimentarius Commission (CAC) decided in 2013, at the request of India, to establish the Codex Committee on Spices and Culinary Herbs (CCSCH). The main task of the Committee is to develop global quality standards for spices and culinary herbs in order to facilitate the flow of the above-mentioned goods and to ensure fair trade practices. The Committee decided to start work on standards for black, white, and green pepper, cumin, oregano, and thyme as a first step [78]. 

Another legal act aimed at improving food safety, as well protecting against food fraud is Commission Implementing Regulation (EU) 2019/1715 of 30 September 2019 laying down rules for the functioning of the information management system for official controls and its system components (the IMSOC Regulation). The regulation establishes an “alert and cooperation network” consisting of the Rapid Alert System for Food and Feed (RASFF), the Administrative Assistance and Cooperation (AAC),and the “food fraud network”, composed of the Commission, Europol, and the liaison bodies designated by EU member states [79].

The communication established between these networks facilitates cooperation and rapid reaction to unfair practices, which are increasingly frequent every year, on the EU food market.

On 1 July 2020, the Act of 23 January 2020 amending the Act on the commercial quality of agricultural and food products and certain other acts came into force in Poland [80]. This act establishes the merger of the tasks of two inspections that have so far controlled the quality of food trade in Poland. A single inspection—Commercial Quality Inspection of Agricultural and Food Products (IJHARS)—will be responsible for the supervision of the commercial quality of all such products, taking over the tasks of the Trade Inspection in the field of food control. According to the amendment of the discussed act, the inspection is established to control compliance with the provisions on the commercial quality of agricultural and food products, including the protection of the interests and rights of final consumers. Entrusting all tasks related to the supervision of the commercial quality of agricultural and food products to one specialized official control body ensures more effective response to market irregularities. It is expected to contribute to the faster elimination of non-compliant quality products from the market and a better use of the potential of the combined inspection services and laboratory facilities.

In keeping with the organizational changes in the European and Polish food control systems, the official control procedures have also changed. On 27 April 2019, Regulation (EU) 2017/625 of the European Parliament and of the Council of 15 March 2017 on official controls and other official activities performed to ensure the application of food and feed law, rules on animal health and welfare, and plant health and plant protection products came into force [81].

On 2 April 2021, the Commission Regulation (EU) 2019/649 of 24 April 2019 amending Annex III to Regulation (EC) No 1925/2006 of the European Parliament and of the Council as regards trans-fat, other than trans-fat naturally occurring in fat of animal origin, came into force. This regulation states the maximum permissible content of synthetic trans fats in food products as 2 g per 100 g of fat [82].

All aforementioned legal acts also apply to herbs, spices, and spice mixes. Good legal practices allow for proper supervision over the commercial quality of agricultural and food products and contribute to the faster elimination of non-compliant products from the market

## 6. Conclusions

In order to succeed on the market, every enterprise must adopt an operating strategy that will not only ensure its survival on the market, but also the possibility of effective development under the conditions of market competition. One of the possible strategies for operating on the market is the continuous improvement of products through innovation. The key factor of innovative solutions is the accumulation of appropriate knowledge resources. In this study, the authors focused on innovative food products from the group of spices and seasoning mixes. The analysis carried out on the basis of the results of IJHARS scheduled inspections, notifications in the RASFF system, and the results of own research showed insufficient use of knowledge resources in the field of product and technological innovations in spices and seasoning blends. A large number of notifications in this group of food products indicate that ensuring the safety of spices and seasoning mixes should be aimed at raising awareness and knowledge, primarily in the field of sourcing and processing of the raw material for spices, as well as commodity knowledge in terms of ensuring safety and quality systems in food production. In order to raise the quality of spice products, it is necessary to increase the knowledge of employees as a resource of the organization, increase cooperation with scientific organizations, and manage the company in accordance with the knowledge-based enterprise management strategy.

## Figures and Tables

**Table 1:** Number of notifications of pollutants in herbs and spices in the EU Rapid Alert System for Food and Feed (RASFF) in 2015–2019 based on the RASFF-EC-Notifications list.

Year	Notifications	Border Rejections	Official Market Control
2015	158	72	34
2016	195	110	39
2017	150	78	38
2018	124	42	50
2019	209	104	72

**Table 2:** Selected notifications about microbiological threats and radiation treatment in spices originating from different countries in 2015–2019 based on the RASFF-EC-Notifications list.

Notification Reason	Spice	Country of Origin
Aflatoxin B1, ochratoxin A	Nutmeg	Indonesia
	Spice mix	Sri Lanka, Bangladesh
	Ginger	India
	Spice mix, dried pepper	Ethiopia
	Kebab spice mix	Ghana
	Black pepper	Vietnam
	Chili	India, China, Pakistan, Vietnam, Bangladesh
	Spice mix	Kuwait, Hong Kong
	Dried pepper	Turkey, India, Peru
*Salmonella* sp.	Cumin	Jordan
	Turmeric	Peru
	Nettle powder	Albania, Bulgaria
	Smoked pepper	Spain
	Spice mix	Thailand
	Ginger	Nigeria
	Black pepper	Brazil
	Spice mix	Spain, Austria, Serbia
	Chili	Vietnam
*Escherichia coli* Shiga toxins	Pepper	Vietnam
	Coriander	Vietnam
	Perilla (*Perilla frutescens*)	Laos
	Basil	United Kingdom
	Fresh mint	Laos
Non-declared radiation treatment of spices	Garlic powder	China
	Coriander powder	Bangladesh
	Ginger	China
	Dried chives	China
	Nutmeg	India

**Table 3:** Selected notifications due to the presence of undeclared additives, pesticides, and plant protection products in spices from different countries in 2015–2019 based on the RASFF-EC-Notifications list.

Notification Reason	Spice	Country of Origin
Non-declared addition of colorants or other substances (e.g., sodium benzoate)	Spice mix	Pakistan
	*Sumac* spice	Iran
	Dried pepper	Ghana
	Spice mix	Thailand
	Chili	India
Presence of crop protection chemicals and pesticides	Mint leaves	Israel
	Curry	India
	Basil	Laos, Cambodia, Thailand
	Chili peppers	Gambia
	Ginger	Nicaragua
	Chili	Dominican Republic, Vietnam
	Cardamom, cumin	India
	Fresh mint	Morocco
	Dried parsley	Egypt
	Fennel seeds	Egypt
	Coriander	Thailand
	Dried pepper	Spain
	Cumin	India

## Data Availability

The datasets generated for this study are available on request to the corresponding author.
